# Alternative stopping rules to limit tree expansion for random forest models

**DOI:** 10.1038/s41598-022-19281-7

**Published:** 2022-09-06

**Authors:** Mark P. Little, Philip S. Rosenberg, Aryana Arsham

**Affiliations:** 1 Radiation Epidemiology Branch, National Cancer Institute, Bethesda, MD 20892-9778 USA; 2 Biostatistics Branch, National Cancer Institute, Bethesda, MD 20892-9778 USA; 3 Integrative Data Analytics Program, Center for Data, Mathematical & Computational Sciences, Goucher College, Baltimore, MD USA; 4 Radiation Epidemiology Branch, Division of Cancer Epidemiology and Genetics, Department of Health and Human Services, National Cancer Institute, National Institutes of Health, 9609 Medical Center Drive, Bethesda, MD 20892-9778 USA

**Keywords:** Metabolic disorders, Prognostic markers, Computational science, Computer science, Software, Statistics

## Abstract

Random forests are a popular type of machine learning model, which are relatively robust to overfitting, unlike some other machine learning models, and adequately capture non-linear relationships between an outcome of interest and multiple independent variables. There are relatively few adjustable hyperparameters in the standard random forest models, among them the minimum size of the terminal nodes on each tree. The usual stopping rule, as proposed by Breiman, stops tree expansion by limiting the size of the parent nodes, so that a node cannot be split if it has less than a specified number of observations. Recently an alternative stopping criterion has been proposed, stopping tree expansion so that all terminal nodes have at least a minimum number of observations. The present paper proposes three generalisations of this idea, limiting the growth in regression random forests, based on the variance, range, or inter-centile range. The new approaches are applied to diabetes data obtained from the National Health and Nutrition Examination Survey and four other datasets (Tasmanian Abalone data, Boston Housing crime rate data, Los Angeles ozone concentration data, MIT servo data). Empirical analysis presented herein demonstrate that the new stopping rules yield competitive mean square prediction error to standard random forest models. In general, use of the intercentile range statistic to control tree expansion yields much less variation in mean square prediction error, and mean square prediction error is also closer to the optimal. The Fortran code developed is provided in the Supplementary Material.

## Introduction

Breiman developed the idea of bootstrap aggregation (bagging) models^1^, commonly used with bootstrap averages of tree models, as a way of flexibly modeling data. Bootstrap averaging is a way of reducing the prediction variance of single tree models. However, correlations between trees implied that there would be limits to the reduction in prediction errors achieved by increasing the number of trees. The random forest (RF) model was developed by Breiman^2^ as a way of reducing correlation between bootstrapped trees, by limiting the number of variables used for splitting at each tree node. RF models often achieve much better prediction error than bagging models. RF models have proved a straightforward machine learning method, much used because of their ability to provide accurate predictions for large and complex datasets and availability in many software packages. The semi-parametric model is determined by three user specified parameters, one of the more critical being the stopping criterion for node splitting, the minimum node size of each potential parent node. The node size regulates the model complexity of each tree in the forest and has implications on the statistical performance of the algorithm. In a recent paper Arsham et al.^3^ proposed using as stopping criteria the size of the offspring nodes and showed in a series of simulation studies circumstances in which performance over a standard RF model could be improved in this way.

The original RF algorithm by Breiman^2^ used the minimum size of the parent node to limit tree growth. This implementation of the RF algorithm has been utilized in several packages including the randomForest^4^ and ranger^5^ packages; ranger^5^ appears to be among the most efficient implementation of the standard RF algorithm. The problem of how to select the node size in RF models has been much studied in the literature^6,7^. There are a number of available packages that allow for alternatives to the standard parental node size limit for node splitting. In particular the randomForestSRC^8^ and the partykit^9,10^ R packages both allow for splits to be limited by the size of the children nodes.

In this short paper we outline a number of variant types of RF algorithms, generalizations of the RF model developed by Breiman^2^, and which use a number of different criteria for stopping tree expansion, in addition to the canonical ones of Breiman^2^ and Arsham et al.^3^. We illustrate fits of model to the National Health and Nutrition Examination Survey (NHANES) data and four other datasets, the Tasmanian Abalone data, the Boston Housing crime rate data, the Los Angeles ozone concentration data, and the MIT servo data; these last four datasets are all as used in the paper of Breiman^2^. Further description of the data is given in Table 1.

**Table 1:** Description of five datasets fitted.

Dataset	NHANES	Tasmanian abalone	Boston housing	Los Angeles ozone	MIT servo
Number of datapoints	8343	4177	506	330	167
Dependent variable	Glycohemoglobin (mg/dL)	Rings ( = age)	Crime rate per capita by town	Upland CA maximum ozone	Rise time of servo
Explanatory variables	Age (years)	Sex (M/F)	Proportion of residential land zoned for lots over 25,000 sq ft	Vandenberg 500 mb height	Type of motor linkage (A,B,C,D,E)
	Weight (kg)	Length (mm)	Proportion of non-retail business acres per town	Wind speed (mph)	Type of screw linkage (A,B,C,D,E)
	Systolic blood pressure (mm Hg)	Diameter (mm)	Charles River variable ( = 1 if tract bounds river, 0 otherwise)	Humidity (%)	Gain setting 1
	Diastolic blood pressure (mm Hg)	Height (mm)	Nitric oxides concentration (parts per 10^7^)	Sandburg AFB temperature	Gain setting 2
	Glucose (mg/dL)	Whole weight (g)	Average number of rooms per dwelling	Inversion base height	
	Cholesterol (mg/dL)	Shuck weight (g)	Proportion of owner-occupied units built prior to 1940	Daggot pressure gradient	
	Triglycerides (mg/dL)	Viscera weight (g)	Weighted distances to five Boston employment centres	Inversion base temperature	
	Urination (minutes between last urination)	Shell weight (g)	Index of accessibility to radial highways	Visibility (miles)	
	Sedentary activity (minutes of sedentary activity in typical day)		Full-value property-tax rate per $10,000	Day of year	
	Gender		Pupil-teacher ratio by town		
	Race		1000 × (proportion blacks (by town) − 0.63)^2^		
	Risk for diabetes (ever been told you have health risk for diabetes)		% lower status of the population		
	Kidneys (ever been told you had weak/failing kidneys)		Median value of owner occupied homes in $1000s		
	Stroke (ever been told you had a stroke)				
	Weight loss (doctor told you to control/lose weight)				
	Salt (doctor told you to reduce salt in diet)				
	Cigarette smoking (used any tobacco product in last 5 days)				
	Income				
	Night urination (how many times urinate in night)				
	Year (2016, 2018)				

## Results

**Figure 1:**
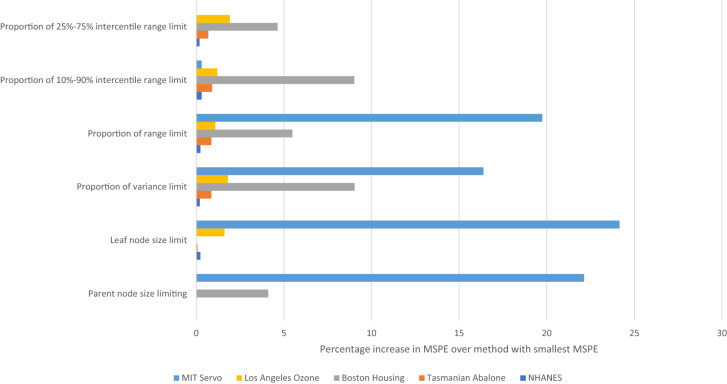
Percentage increase in mean square predictive error (MSPE) for each stopping rule over the tree expansion rule yielding lowest MSPE, for each dataset.

As can be seen from Table 2 and Fig. 1, for the NHANES, Tasmanian Abalone and Los Angeles Ozone datasets the default (parent node size) tree-expansion limitation yields the lowest mean square prediction error (MSPE), although in all cases the MSPE is very close for most other tree-expansion limitation statistics. In particular the MSPE using leaf-node limitation is within 2% of that for parent node limitation. However, for the Boston Housing data leaf-node limitation yields an MSPE that is substantially better, by about 4%, than parent-node limitation, and indeed any other method of tree-limitation. The MSPE using 25–75% intercentile range limitation is substantially better than any for the MIT servo data, the only other method that works nearly as well uses 10–90% intercentile range. All other methods of tree-expansion limitation, in particular both leaf-node and parent-node methods, have MSPE that is at least 15% larger (Table 2). In general use of the two intercentile range statistics (intercentile 10–90% range, intercentile 25–75% range) to control tree expansion yield much less variation in MSPE; in particular, using the 25–75% range, the MSPE does not exceed 5% of the MSPE for the best tree-expansion method for each dataset (Fig. 1).

**Table 2:** Measures of goodness of fit (mean square cross-validated test error) to glycohemoglobin percentage, estimated from hold-out test set (2017–2018 NHANES data) associated with fit of random forest model fit to 2015–2016 NHANES data, and similar measures of goodness of fits to Tasmanian Abalone data, Boston Housing data, Los Angeles Ozone data and MIT Servo data.

Method of limiting tree growth	NHANES	Tasmanian abalone	Boston Housing	Los Angeles ozone	MIT servo
Parent node size limiting	**0.1395**	**4.5088**	32.2552	**15.6340**	0.2729
Leaf node size limit	0.1398	4.5119	**30.9823**	15.8862	0.2774
Proportion of variance limit	0.1398	4.5475	33.7808	15.9173	0.2601
Proportion of range limit	0.1398	4.5475	32.6826	15.8043	0.2676
Proportion of 10–90% intercentile range limit	0.1399	4.5497	33.7754	15.8223	0.2241
Proportion of 25–75% intercentile range limit	0.1397	4.5398	32.4181	15.9343	**0.2235**

The optimal model for each method of tree-growth limitation is shown in boldface.

## Discussion

We have presented a number of alternative tree-expansion stopping rules for RF models. It appears that for some datasets, in particular the NHANES, Tasmanian Abalone and Los Angeles Ozone data the new types of stopping rules that we fit have very similar MSPE as the standard stopping rules normally used by RF models (Table 2, Fig. 1). However, for two other datasets, the Boston Housing and MIT Servo data, it is clear that two particular variant stopping rules fit substantially better than the standard RF model (Table 2, Fig. 1). In general, use of the intercentile 25–75% range statistic to control tree expansion yields much less variation in MSPE, and MSPE also closer to the optimal. The MSPE for this measure does not exceed 5% of the MSPE for the best tree-expansion method for each dataset (Fig. 1).

One of the parameters in the RF algorithm is the minimum size of the node below which the node would remain unsplit. This is very commonly available in implementations of the RF algorithm, in particular in the randomForest package^4^. The problem of how to select the node size in RF models is much studied in the literature. In particular Probst et al.^7^ review the topic of hyperparameter tuning in RF models, with a subsection dedicated to the choice of terminal node size. This has also been discussed from a more theoretical point of view in a related article by Probst et al.^6^. As Probst et al. document, the optimal node size is often quite small, and in many packages the default is set to 1 for classification trees and 5 for regression trees^7^. There are a number of packages available that allow for alternatives to the standard parental node size limit for node splitting. In particular the randomForestSRC^8^ and the partykit^9,10^ R packages both allow for splits to be limited by the size of the offspring node. As far as we are aware no statistical package uses the range, variance or centile range based limits demonstrated here. It should be noted that the use of limits of parental and offspring node size are not equivalent. While it is obviously the case that if the offspring nodesize is at least $n$ then the parental node size must be at least $2n$, the reverse is clearly not the case. For example, it may be that among the candidate splits of a particular node of size $2n$ would in general be offspring nodes of sizes $1,2,...,n - 1,n,n + 1,...2n - 1$. Were one to insist on terminal nodes being of size $n$ then only the split into two nodes each of size $n$ would be considered, whereas without restriction on the size of the terminal nodes potential candidates would in general include nodes of size $1,2,...,n - 1,n + 1,...2n - 1$ also, although the splitting variables might not in general allow all these to occur.

Numerous variants of the RF model have been created, many with implementations in R software. For example, quantile regression RF was introduced by Meinshausen^11^ and combines quantile regression with random forests and its implementation provided in the package quantregForest. Garge et al.^12^ implemented a model-based partitioning of the feature space, and developed associated R software mobForest (although this has now been removed from the CRAN archive). Seibold et al.^13^ also used recursive partioning RF models which were fitted to amyotrophic lateral sclerosis data. Seibold et al. have also developed software for fitting such models, in the R model4you package^14^. Segal and Xiao^15^ have outlined use of RFs for multivariate outcomes and developed the R MultivariateRandomForest package^16^ for fitting such models. A number of more specialized RF algorithms have also been developed. Wager and Athey^17^ used concepts from causal inference, and introduced the idea of a causal forest. Foster et al.^18^ also used standard RFs as part of a causal (counterfactual) approach for subgroup identification in randomized clinical trial data. Li et al.^19^ have applied more standard RF models to analyze multicenter clinical trial data. An algorithm that combines RF methods and Bayesian generalized linear mixed models for analysis of clustered and longitudinal binary outcomes, termed the *binary mixed model forest* was developed by Speiser et al.^20^, using standard R packages. Quadrianto and Ghahramani^21^ also proposed a novel RF algorithm incorporating Bayesian elements, which they implemented in Matlab, and compared this model with a number of other machine learning approaches in analysis of a number of datasets. Ishwaran et al.^22^ outlined a survival RF algorithm that is applicable to right-censored survival data; an R package randomSurvivalForestSRC (now removed from the CRAN repository) has been written implementing this model, among other time-to-event RF variants. For genomic inference two R packages implementing standard RF models have been developed by Díaz-Uriarte and de Andrés^23^ and Diaz-Uriarte^24^, GeneSrF and varSelRF. RF have been used in meta-analysis, and a software implementation is provided by the R package metaforest^25^. The grf:geographical random forest package of Georganos et al.^26^ provides an implementation of the RF model specifically aimed at geographical analyses.

Our principal focus has been on improvement in prediction error, as measured by MSPE. Attempts have been made to reduce the bias in RF models, a related but different problem. Zhang and Lu^27^ outlined five different methods of doing this. Song outlined a different method of bias correction, via residual rotation^28^. Reducing bias is obviously important, although machine learning methods often prioritize reduction in prediction error, even at the cost of introducing a small amount of bias^29^. In principle it would be possible, although in some cases computationally irksome, to ascertain uncertainties in MSPE using a double bootstrap.

We have outlined stopping rules with specific application to regression trees. However, the basic idea would obviously easily carry over to classification trees, using for example the Gini or cross-entropy loss functions.

## Methods

### Data

The NHANES data that we use comprises data for the 2015–2016 and 2017–2018 screening samples, the former used to train the RF and the latter as test set. There are *n* = 4292 individuals in the 2015–2016 data, and *n* = 4051 individuals in the 2017–2018 data. A total of 19 descriptive variables (features) are used in the model, with laboratory glycohemoglobin percentage as the outcome variable, a continuous measure. The population weights given in these two datasets are used to weight mean square error (MSE). The version of the NHANES data is exactly as used in the paper of Arsham et al.^3^. We also employ four other datasets, the Tasmanian Abalone data, the Boston Housing crime rate data, the Los Angeles ozone concentration data, and the MIT servo data; these last four datasets are all as used in the paper of Breiman^2^. A description of all these datasets is given in Table 1. The five datasets are all given in Supplement S1.

### Statistical methods

There are minimal adjustable parameters in the standard RF algorithm^2^, specifically the number of trees (i.e. the number of bootstrap samples, **ntree**), and the number of variables sampled per node (**mtry**) used to determine the growth of the tree, and the maximum number of nodes per tree (**maxnodes**). The version of the algorithm that we have implemented incorporates a number of additional parameters that determine whether tree generation is halted, specifically:

- The proportion of the total variance (in the total dataset) of the outcome variable in a given node used to determine whether to stop the further development of the tree from that node downwards;
- The proportion of the total range (= maximum − minimum) (in the total dataset) of the outcome variable in a given node used to determine whether to stop the further development of the tree from that node downwards;
- The proportion of the intercentile range [X%, 100 − X%] (in the total dataset) of the outcome variable in a given node used to determine whether to stop the further development of the tree from that node downwards. We used X = 10% and X = 25%.
- The minimum number of observations per parent node.
- The minimum number of observations per terminal (leaf) node.

The tree generation at a particular node is halted if any of conditions (a)–(e) is triggered. In most implementations of the standard RF model^2^, for example the R randomForest package^4^, only criteria (d) is available; in some software, in particular in the randomForestSRC^8^ and partykit^9^ R packages criteria (d) and (e) are available as options. The paper of Arsham et al.^3^ outlined the use of criterion (e) in the context of regression trees. Table 2 outlines the minimum mean square prediction error (MSPE) obtained using the 2017–2018 NHANES data as test set, with model training via the 2015–2016 data. For all other datasets MSPE was defined via tenfold cross validation. In all cases MSPE was the minimum value using **ntree** = 1000 trees with **maxnodes** = 1000. We employed a number of sampled variables per node **mtry** generally about half the total number of independent variables, so **mtry** = 10, 4, 7, 5, 2, for the NHANES, Tasmanian Abalone, Boston Housing, Los Angeles Ozone and MIT Servo datasets, respectively.

In all cases the categorical variables are treated simply as numeric (non-categorical) variables. We also performed additional model fits in which we used Breiman’s method of coding categorical variables^2^, but as these generally yielded inferior model fits, as measured by the **minMSPE**, we do not report these further.

The Fortran 95-2003 code implementing the regression random forest algorithm described above is given in Supplement S1, along with a number of parameter steering files for the five datasets fitted.

### Ethics declaration

This study has been approved annually by the National Cancer for Health Statistics Research Ethics Review Board (ERB), and all methods were performed in accordance with the relevant guidelines and regulations of that ERB. All participants signed a form documenting their informed consent, and participants gave informed consent to storing specimens of their blood for future research.

## Supplementary Information

Supplementary Information.

## Data Availability

The National Health and Nutrition Examination Survey data is freely available for download from https://wwwn.cdc.gov/nchs/nhanes/continuousnhanes/default.aspx?BeginYear=2015 (2015–2016 data) and https://wwwn.cdc.gov/nchs/nhanes/continuousnhanes/default.aspx?BeginYear=2017 (2017–2018 data).
