# Supplementary material for: Alternative stopping rules to limit tree expansion for random forest models
Source: Sci Rep. 2022 Sep 6;12:15113. doi: 10.1038/s41598-022-19281-7 (PMC9448733; doi:10.1038/s41598-022-19281-7)
Supplement: Supplementary file 1 — Supplementary Information. [file 41598_2022_19281_MOESM1_ESM.zip › Index to Supplement S1.docx]

**Supplement S1**

**Fortran program**

fitter_random_forest.for

**Windows batch file to run fortran program**

run8.bat

**Datasets used for paper**

1. National Health and Nutrition Examination Survey (NHANES) 2015-2018

NHANES 2015-2018 combined.xlsx

NHANES_2015_2018_combined.lis

1. Tasmanian Abalone data

Tasmanian Abalone.xlsx

Tasmanian_Abalone.lis

1. Boston Housing crime rate data

Boston housing.xlsx

Boston_housing.lis

1. Los Angeles ozone concentration data

Los Angeles Ozone.xlsx

Los_Angeles_Ozone.lis

1. MIT servo data

MIT Servo.xlsx

MIT_Servo.lis

**Steering input files to control running of fortran program**

fit_random_forest*.inp
